# Developing a consensus of recovery from suicidal ideations and behaviours: A Delphi study with experts by experience

**DOI:** 10.1371/journal.pone.0291377

**Published:** 2023-09-20

**Authors:** Esmira Ropaj, Gillian Haddock, Daniel Pratt

**Affiliations:** 1 Division of Psychology & Mental Health, Manchester Academic Health Science Centre, School of Health Sciences, University of Manchester, Manchester, United Kingdom; 2 Greater Manchester Mental Health NHS Foundation Trust, Manchester Academic Health Science Centre, Manchester, United Kingdom; University of Bologna: Universita di Bologna, ITALY

## Abstract

**Background:**

Understanding recovery in mental health has received significant attention and consequently, recovery has been incorporated into health policy across many countries in the Global North. In comparison, the concept of ‘recovery’ from suicidal thoughts and behaviours has received little attention. However, the few studies in this area appear to suggest that recovery is a complex and an idiosyncratic process with many contributing factors. This can present a challenge for clinicians and services seeking to become more recovery focused. Thus, it seems of importance to develop a consensus on how recovery from suicidal thoughts and behaviours is conceptualised.

**Aim:**

The study aimed to use the Delphi design to establish a consensus of how recovery is defined by those with lived experience of suicidal thoughts and behaviours. The Delphi method draws on the expertise of a panel, often involving clinicians, researchers and lived experience experts to develop consensus over a topic by inviting them to rate the importance of, often a series of statements to a given topic area.

**Method:**

Lived experience experts were asked to complete two rounds of questionnaires distributed online to capture their views on recovery.

**Results:**

A total of 196 individuals gave their views on the first round of the study and 97 gave their views on the second round. A final list of 110 statements was developed that 80% or more of participants defined as essential or important. Statements covered items that were important in defining, facilitation and hindering the process of recovery.

**Conclusion:**

Findings are consistent with the wider literature that suggests that recovery is an idiosyncratic process, but with many commonly shared features. Here we also show that a comprehensive definition of recovery must include factors that hinder the process of recovery. Implications and recommendations for practice, policy development and future research are discussed.

## Introduction

Suicide is one of the leading causes of death globally, with the World Health Organization (WHO) reporting that over 700,000 individuals died by suicide in 2019 [1]. The reduction of the number of deaths by suicide has been a key focus for governments globally, including the United Kingdom (UK). In the UK, national prevention efforts have focused, amongst others, on reducing access to means and targeting at risk groups (e.g., mental health service users, young and middle-aged men; [2]). In line with National Institute for Health and Clinical Excellence guidelines for suicide prevention [3], healthcare services (e.g., mental health inpatient services) have also focused on reducing access to means as an approach to supporting individuals. With the aim of supporting prevention efforts, a great body of research has sought to identify risk factors for suicide. Little attention has, however, been given to recovery from suicidal thoughts and behaviours, despite the fact that having an understanding of the lived experience of recovery could contribute to ongoing suicide prevention efforts [4].

Supported by the service user movement, recovery has become the cornerstone of mental health policy and practice within the Global North [5, 6]. The implementation of the recovery model has thus resulted in a number of important shifts, including the prioritisation of service user lead definitions of recovery when providing support. Whilst recovery is understood to be an idiosyncratic process and competing definitions (clinical vs service user) have been proposed, a range of models have been developed (e.g., CHIME; [7]). These models highlight that there are common features of recovery shared across individuals, including, connection, hope, empowerment, and meaning in life, to name a few [7–9]. This line of research enquiry has facilitated the production of guidelines and tools for developing recovery focused services and practices within mental health [10, 11]. In comparison, the development of the recovery literature in the context of suicidal thoughts and behaviour has been much slower.

For those experiencing thoughts about ending their life, the primary focus of psychological intervention, to date, has been on reducing suicidal ideation and behaviours. Indeed, research defines clinical recovery as a shift in scores from a clinical to a non-clinical range on a number of measures which capture suicidal ideation and suicidal probability [12–14], with many intervention studies demonstrating recovery through a reduction in the occurrence of suicidal thoughts and behaviours [14–16]. Furthermore, studies exploring the experience of recovery include individuals who are no longer experiencing suicidal thoughts or behaviours [17, 18]. This highlights that a reduction in suicidal thoughts and behaviours is an important indicator of recovery. In this sense, recovery is defined as an endpoint to be arrived at rather than a journey towards wellness. We know, however, that interventions do more than simply help individuals to reduce the frequency of suicidal ideations and behaviours (e.g., support the development of positive relationships with others). We therefore need a more holistic way to capture recovery from suicidal thoughts and behaviour that extends beyond a reduction in symptoms.

Developing an understanding of recovery that extends beyond symptoms reduction is likely to be of value to professionals (e.g., mental health inpatient services staff), who often report uncertainty around how to approach conversations about suicide with individuals under their care, fearing that doing so might cause the individual distress or harm [19]. Furthermore, staff can often feel unsure about how best to support suicidal individuals; thus, the focus of clinical input is often on identifying risks of suicidal behaviour and seeking to reduce or remove the behaviour [20, 21]. In this sense, suicide prevention reflects a ‘risk-dominant’ paradigm [22], the usefulness of which has been questioned within the literature [23]. Furthermore, we know that even when suicide specific psychological interventions are provided, many individuals continue to experience suicidal thoughts and that this need not necessarily indicate a negative outcome [24]. The focus on clinical recovery may therefore leave many service users with inadequate support and maybe cause further distress in those not ‘recovering’. Awenat et al. [25] note that service users want to develop an understanding of their suicidal thoughts and want to explore factors that help them to overcome suicidal thoughts and behaviours. Seeking to understand the individual’s story has been promoted by a number of research studies [26, 27]. Clinicians may therefore benefit from being able to provide recovery orientated support that draws on the subjective experience of those who have had and continue to have suicidal thoughts and engage in suicidal behaviours.

Qualitative studies exploring participants’ experience of psychological interventions highlight that as well as a reduction in the frequency or severity of thoughts about suicide, recovery can also be about developing a sense of hope, working towards personal goals, and developing positives ways of thinking about the self and the future [24, 28, 29]. Others have highlighted that recovery can also be about building a life with meaning, feeling empowered and taking control over one’s recovery [18, 30–32]. Importantly the literature appears to suggest that recovery is an ongoing process, where the individual is able to engage with life at their own pace whilst also continuing to experience suicidal thoughts and behaviours [17, 33, 34]. Adding to the complexity of seeking to define recovery, a small body of literature has found that a range of factors are important in facilitating recovery. These factors include, connecting with supportive others (e.g., family, professionals), setting goals for the future, reconnecting with spirituality and the self [32, 35, 36], and developing problem-solving and emotional regulation skills have consistently been identified [35, 37].

The multiple definitions of recovery arising from exploring the lived experience of suicidal thoughts and behaviours is reflective of the multiple models and definitions of recovery that exist in the wider literature on the concept of recovery [8, 9, 38], which all highlight the idiosyncratic nature of recovery. This along with the different competing perspective on recovery that currently exist between clinical and non-clinical approaches as well as the multitude of factors said to facilitate recovery can present a challenge to services seeking to be recovery orientated, making it difficult to determine what such a service looks like and to evaluate how effective the service is in terms of supporting people to recover. Furthermore, these factors can make it difficult for professionals to know how best to support individuals who are experiencing suicidal thoughts or behaviours. A better understanding of recovery, and the factors that are essential in supporting this process, could make an important contribution to the development of suicide prevention interventions. As well as this, such knowledge can also contribute to the development of a ‘recovery from suicide’ measure, which would allow us to assess the effectiveness of interventions beyond outcomes focused on occurrences of suicidal thoughts and behaviours. An important next step in developing the recovery literature is to bring together this literature with the aim of developing a consensus on what recovery from suicide looks like and what factors may help recovery.

Overall, no study has so far sought to develop a consensus of what is meant by recovery and what factors facilitate recovery in the context of suicidal thoughts and behaviours. While it is anticipated that conceptualisations of recovery from suicidal thoughts and behaviours will be unique to each individual, there are likely to be common factors that are significant in supporting recovery [30]. Investigating the extent to which those with lived experience agree about what constitutes recovery from suicidal thoughts and behaviours and what facilitates this process therefore feels important, particularly because it is yet to be investigated.

Within the current study, the research team drew on the Delphi method to develop a consensus. The Delphi design is a systematic method that seeks to involve a panel of ‘experts’ who, over a series of two or more rounds, are invited to rate a number of items in relation to their importance to a given topic. In line with the existing recovery literature that argues for and privileges service user definitions of recovery [10], the current study aimed to recruit individuals with lived experience of suicidal thoughts and behaviours in order to develop an understanding of how we define recovery from suicidal ideation and behaviour and what factors are important in helping people to recover.

## Method

### Delphi expert consensus method

A modified e-Delphi Method [39] was used to arrive at a consensus about what constitutes recovery from suicidal ideation and behaviour and what factors facilitate this process of recovery. Such a method is suitable for the study of a research topic where there is a lack of clarity and debate [40]. The e-Delphi format allowed the research team to reach individuals across the country and supported the engagement of individuals who may normally not engage in research as a result of stigma [41]. Consistent with research that has sought to define recovery using the Delphi method [42], the Delphi process in the current study began by carrying out a systematic search of peer reviewed and grey literature to identify key concepts relating to the topic of interest. From this, a series of statements were then produced by the research team. These were reviewed and adapted by an expert panel of individuals with lived experience of suicidal thoughts and behaviours. The research team then identified expert panel members, following which the Delphi consensus ratings by expert panel members took place. A final list of statements pertaining to the conceptualisation of recovery was collated. The development of the Delphi questionnaire was not informed by a specific model of recovery. Instead we adopted a holistic model of recovery that was informed by service user experiences reported in the literature [31, 32]. Furthermore, given that multiple definitions of recovery exist within the literature; from user-based definition that focus on social functioning [43] to clinical definitions that focus on clinical outcomes [44], being informed by a single model would likely result in the exclusion of important aspects of recovery. The study was approved by an NHS Research Ethics Committee (21/NW/0065).

### Systematic search of the literature

#### Search of the peer reviewed literature

Consistent with guidance on developing Delphi studies in mental health research [45], a systematic search of existing literature was carried out to identify content pertinent to the conceptualisation of recovery from suicidal thoughts and behaviours. The research team focused on content that related to how we might define recovery as well as factors that are important in helping individuals to recover. Databases PsychINFO, Medline, and CINAHL were searched from the point of conception to February 2020 using the following search terms: Recover* OR “protective factors” OR “reasons for living” AND suicid*. Author ER then screened titles and abstracts for eligibility, and then read through full texts and extracted information pertaining to factors that support recovery and that define recovery. Dissertations, reports and conference posters were all included.

#### Search of grey literature

The above was supplemented by a search of the grey literature. Online dissertation repositories (e.g., EThOS) were searched using the above search terms. Alongside this, an Honorary Research Assistant (an undergraduate psychology student) searched through a number of websites of interest to identify policies, procedures and reports on suicide and suicide prevention. Websites detailing personal accounts of recovery were also searched. A list of the websites used is available as a supplementary file (see S1 File). Where websites provided multiple personal recovery accounts a sample of 20 stories were read through in order to identify information of interest.

#### Eligibility criteria

Sources from the peer review and grey literature were eligible for inclusion if they included: i) statements that described how individuals with lived experience of suicidal thoughts and behaviours defined recovery; and ii) statements that described factors which support individuals to recover from suicidal ideation and behaviours. Sources were excluded if they were not in English due to limitations in translation capacity among study authors and if they described conceptualisations of recovery from suicidal thoughts and behaviours in the context of children and young people (i.e., younger than 18 years old).

### Questionnaire development

The extracted text was examined and from this we developed a total of 145 statements. Copies of the statements were then shared with seven individuals with lived experience of suicidal ideation who were paid to consult on the statements. These individuals were recruited from a local research patient and public contributor group. Individuals were instructed to review the statements and make recommendations in terms of the removal, addition or modification of statements. A number of changes were made which included the removal of 53 duplicate statements, the addition of 13 new statements and the modification of the wording of eight statements to improve readability. The above recommendations were reviewed alongside a member of the contributor group. A final list of 105 statements was developed that captured how recovery from suicidal thoughts and behaviours is defined and what supports individuals to recover. The list was then reviewed by the research team for comprehensibility.

### Expert panel

A single panel of individuals with lived experience of suicidal thoughts and behaviours was recruited. The recruitment of ‘experts by experience’ has been encouraged [46], and given the nature of the topic being research, the recruitment of experts by experience as panel members felt most appropriate for a research study that is seeking to develop a consensus on recovery. Lived experience experts have been used as panel members within the existing literature [42, 47]. To be eligible panel members needed to: i) be 18 years or older; ii) speak English in order to respond to statement; iii) be living in the UK; and iv) be currently experiencing or have previously experienced suicidal ideations and/or behaviours within the last 5 years. A timeline of 5 years was used because it felt sufficiently long for individuals to have had experiences of recovery whilst also being able to clearly reflect on what facilitated recovery for them. Panel members were initially also required to provide their GP contact details; however, this was later dropped due to having a significant impact on engagement with the study. Panel members were recruited via convenience sampling through NHS mental health services (such as Early Intervention Services and Community Mental Health Teams), third-sector organisation/voluntary groups and though advertising the study on social media, research websites, university campus and announcement platforms and emails sent to University departments across the UK. Psychology undergraduates were also recruited and received course credits for their participation. Across all recruitment strategies noted above, interested individuals were invited to follow the link within the poster or email and complete the electronic consent form before being sent the questionnaire. Consistent with research that has recruited individuals with lived experience as panel members [42, 48], and in line with evidence which suggest that diversity within the panel strengthens the consensus developed [45, 49, 50], convenience sampling was used in order to reach a broad range of individuals within the timeframe available to the research team. This ensured that consensus was not skewed towards a particular perspective and allowed the research team to reach individuals who were in contact with services as well as those who were not. Being able to do this was important as evidence shows that around 70% of individuals are not in contact with NHS services the year prior to their death by suicide [51].

#### Delphi process

Panel members were asked to complete two rounds of the questionnaire distributed online using a bespoke survey platform built by an eLearning Technologist specifically for use in Delphi studies. A demographic and clinical information sheet, completed prior to the questionnaire link being shared, was used to collect descriptive data on age, gender, ethnicity, length of ongoing difficulties with suicidal thoughts and behaviours, time since last suicidal thought or behaviour and self-reported mental health diagnosis, where applicable. Participants for both rounds were informed that the research team were interested in understanding how we might define recovery from suicidal thoughts and behaviours and what factors were important in supporting people to recover from suicidal thoughts and behaviours. They were instructed to rate each statement in terms of how important it was to a definition of recovery from suicidal thoughts and behaviours or to supporting recovery from these experiences. Each statement was rated on the following 5-point Likert scale: essential, important, unsure/depends, unimportant, should not be included.

#### Round one questionnaire

In the first-round, panel members were presented with the original 105 statements and asked to rate them using the criteria noted above. At the end of the questionnaire, panel members were invited to suggest additional statements. This decision was taken given that recovery is an idiosyncratic process and because what has been written about recovery in the context of suicidal thoughts and behaviour may be limited in scope and may not cover all that is important. As such, inviting panel members to provide additional items aimed to ensure that the resulting list was comprehensive, to allow a more complete consensus to be reached. Furthermore, previous Delphi research has highlighted that panel members should be invited to provide additional items when completing such questionnaires [45], and this approach that has been used in previous Delphi studies [52–55]. The suggested statements were then reviewed by the research team and those which were determined to contribute new ideas not already captured by existing statements were included in the second round. The same approach was followed as for the initial generation of statements, in terms of statements being required to reflect factors that defined or facilitated recovery. In line with existing research [53, 56], statements rated as essential or important by 80% or more of the panel were included as standard. Statements rated as essential or important by 70%-79% of the respondents were re-rated in the second round. Statements not meeting these criteria were excluded from the second round. Round one remained open for four months and the research team took a month to analyse the data and prepare round two.

#### Round two questionnaire

In the second round, which remained opened for a month, panel members were presented with 134 statements (25 statements which needed to be re-rated and 109 new statements generated during round one). The following new statement ’accepting that some days you’ll have thoughts about ending your life and others you won’t’ was rated by 86.60% of participants in round two as essential or important in defining recovery. However, this statement was not included in the final list presented in the results because upon review the research team felt it was similar to two other statements that had already been included in the final list during round one. A number of new statements generated were focused on factors that hinder recovery, which had not been considered in the initial development of the study. However, taking into account factors that hinder recovery appeared to be important to panel members’ conceptualisation of recovery, as such these statements were included in round two. Each panel member was provided with their individual responses as well as the aggregated anonymous results from round one (e.g., percentage of individuals endorsing each statement). In line with previous research and guidelines [45, 55], panel members were instructed to use this information to guide whether they wanted to stick to their original rating or change their mind. Panel members received similar instructions as above with regards to rating each of the statements. As above, statements endorsed as essential or important by 80% or more of the panel were included as standard. All other statements not meeting the above criteria were not considered eligible for inclusion in the final list of statements.

### Analysis

Following the completion of each round, questionnaire responses were analysed using Microsoft Excel [57]. Using the criteria above, each item was assessed to determine the percentage of individuals endorsing items as ‘essential’; ‘important’, ‘unsure/depends’, ‘unimportant’, or ‘should not be included’. Man Whitney- U and Chi-square statistical tests were also carried out to determine whether there were any significant differences between panel members across the two rounds of the questionnaire on key demographic and clinical information (e.g., age, gender, ethnicity, MH diagnosis). Where Chi-square test assumptions were violated the Maximum Likelihood Ratio was reported [58]. The above-mentioned analyses were carried out on SPSS (Version 25.0; [59]). Once the final list of statements was established, they were grouped into three categories: i) factors important in defining recovery, ii) factors important in facilitating recovery and iii) factors that hinder recovery. Specifically, statements that made reference to ‘doing’ were grouped together under factors that facilitate recovery. Statements that encapsulated ‘ways of being’ were grouped together under factors that define recovery. Statements that referred to elements that ‘get in the way of’ recovery were grouped together to form factors that hinder recovery. Finally, the statements were reviewed and grouped into five key themes based on patterns of meaning emerging from each statement.

## Results

A total of 301 individuals consented to take part in the study. One hundred and five individuals did not progress into round one of the study. Reasons for not doing so included not wanting to reveal their identity and thus their suicidal history to their GP, being under the age of 18, errors with email addresses provided and not responding to invites to complete round one. A series of Chi squared tests revealed that there was no significant difference between those who completed the consent form only and those who completed round one, on gender *X*^2^ (5, N = 301) = 3.26, *p* = .659; ethnicity *X*^2^ (13, N = 301) = 17.54, *p* =. 176; length of difficulties *X*^2^ (3, N = 287) = 1.46, *p* =. 691; time since last suicidal thoughts/behaviours *X*^2^ (5, N = 300) = 0.57, *p* = .989; diagnosis *X*^2^ (1, N = 300) = 0.23, *p* = .632. A Mann Whitney U test revealed that there was no significant difference on age between those who completed the consent form only and those who completed round one *U* (*N*_consent only_ = 103, *N*_round one_ = 195) = 9383.50, *z* = -0.93, *p* = .351.

One hundred and ninety-six individuals completed round one and 97 went on to also complete round two. Across round one and round two, the majority of participants were women (64.30% and 67.00%), White British (80.10% and 81.40%) and had a self-reported mental health diagnosis (84.20% and 85.60%). Similarly, across both rounds, the majority of the participants reported that they last experienced suicidal ideation or engaged in suicidal behaviours in the five months prior to completing the study (66.30% and 67.00%). Finally, the most common length of difficulty with suicidal thoughts/behaviour across both rounds was greater than 10 years. Percentages are reported for round one and round two respectively, please see Table 1 for full participant characteristics. Chi squared tests revealed that there was no significant difference between those who completed round one of the study only and those who went on to compete round two on gender (*X*^2^ (5, N = 196) = 4.17, *p* = .746; ethnicity *X*^2^ (11, N = 196) = 14.45, *p* =. 464; length of difficulties *X*^2^ (3, N = 187) = 2.77, *p* =. 443; time since last suicidal thoughts/behaviours *X*^2^ (5, N = 196) = 7.51, *p* = .245; diagnosis *X*^2^ (1, N = 195) = 0.49, *p* =. 554. A Mann Whitney U test revealed that there was no significant difference on age between those who completed round one only and those who completed round two *U* (N _consent only_ = 98, N *round one* = 97) = 4113.00, *z* = -1.63, *p* = .104.

**Table 1:** Participant demographic and clinical information.

	Round 1 (n = 196)	Round 2 (n = 97)
Age—Mean (SD)	31.11 (±10.50)	33.23 (±10.53) *
Gender		
• Male	50 (25.5%)	21 (21.6%)
• Female	126 (64.3%)	65 (67%)
• Non-binary	14 (7.1%)	6 (6.2%)
• Transgender	2 (1.0%)	1 (1%)
• Agender	1 (0.1%)	1 (1%)
• Bigender	1 (0.1%)	1(1%)
• Transfeminine non-binary	1 (0.1%)	1 (1%)
• Transmasculine non-binary	1 (0.1%)	1 (1%)
Ethnicity		
**White**		
English/Welsh/Scottish/Northern Irish/British	157 (80.1%)	79 (81.4%)
Irish	4 (2%)	1 (1%)
Other White background	15 (7.7%)	6 (6.2%)
**Mixed/Multiple ethnic groups**		
White and Black Caribbean	1 (0.1%)	1 (1%)
White and Asian	2 (1%)	2 (2.1%)
**Asian/Asian British**		
British Asian	4 (2.0%)	3 (3.1%)
Indian	3 (1.5%)	2 (6.2%)
Pakistani	3 (1.5%)	1 (1%)
Chinese	3 (1.5%)	-
Other Asian background	1 (0.1%)	1 (1%)
**Black/ African/Caribbean/Black British**		
Black British	1(0.1%)	-
Prefer not to say	2(1%)	1 (1%)
Length of difficulties with suicidal thoughts/behaviour*		
• > 1 year	13 (6.6%)	7 (7.2%)
• 1–5 years	54 (27.6%)	23 (23.7%)
• 6–10 years	54 (27.6%)	24 (24.7%)
• < 10 years	66 (33.7%)	37 (38.1%)
Time since last suicidal thoughts/behaviour		
• 0–5 month	130 (66.3%)	65 (67%)
• 6–12 months	29 (14.8%)	16 (16.5%)
• 13–24 months	18 (9.2%)	9 (9.3%)
• 25–36 months	14 (7.1%)	6 (6.2%)
• 37–48 month	1 (0.1%)	1 (1%)
• 49–60 months	4 (2%)	-
Diagnosis		
• Yes	165 (84.2%)	83 (85.6%)
• No	30 (15.3%)	13 (13.4%)
Prefer not to say	1 (0.1%)	1 (1%)

*Missing data. SD = standard deviation

**Fig 1:**
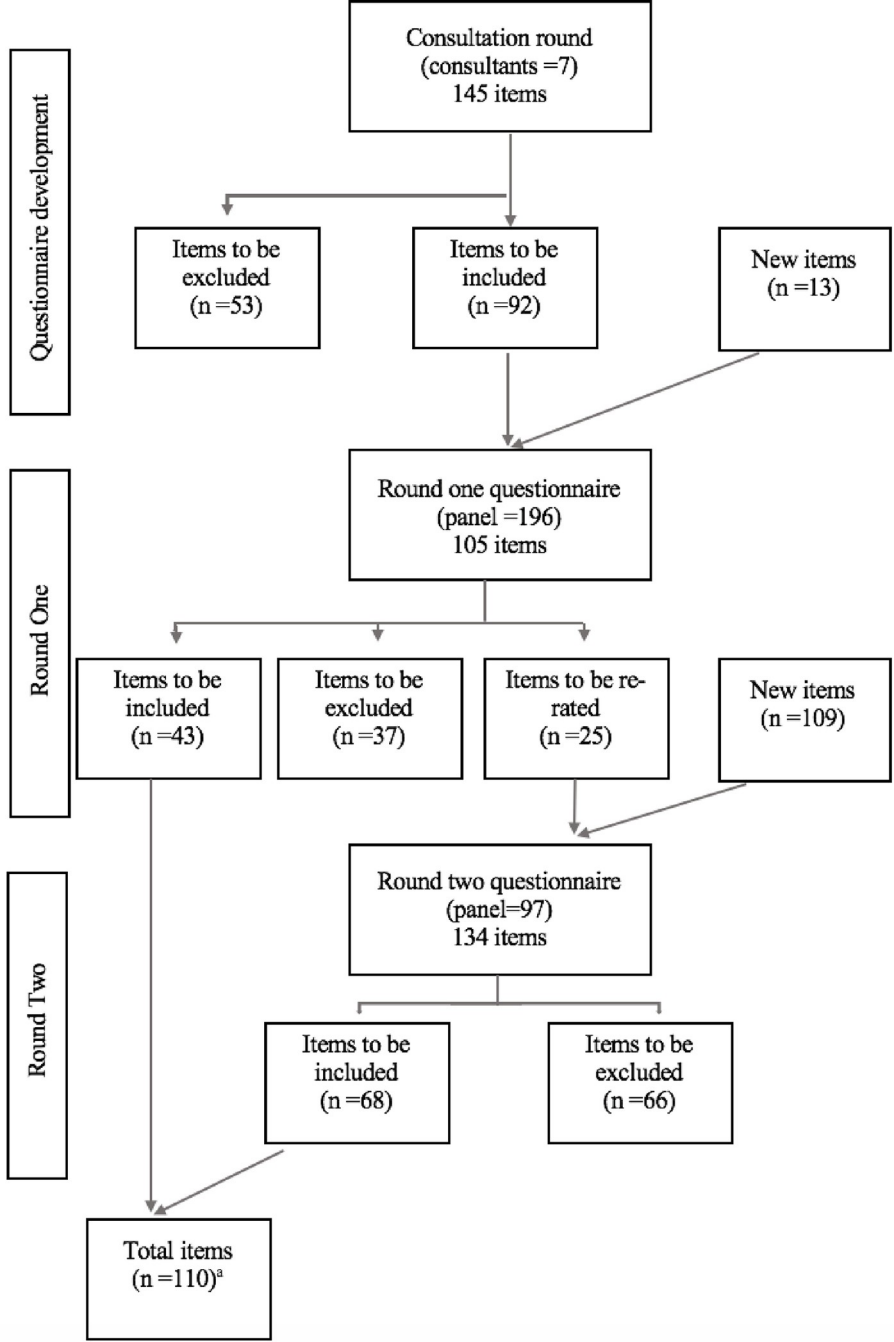
Number of items included, excluded and re-rated across all rounds of the study. ^a^ one item from round two was excluded following data collection due to being similar to two items included in round one.

Fig 1 shows the number of items that were included, excluded and re-rated across both rounds of the study, along with new items generated in round one. A total of 110 items were rated as essential or important by 80% or more of the panel, thus forming part of the final list of statements. The final list of statements was grouped into 3 categories: defining recovery (n = 15), factors that help recovery (n = 87), and factors that hinder recovery (n = 8). Tables 2–4 show these items in their individual category with the percentage of panel members who rated them as essential or important and the round in which the statement was included. The final list of 110 items reflects the idiosyncratic nature of recovery and could highlight a challenge in seeking to identify a homogenous definition of recovery, however it also demonstrates that there are common factors that those with lived experience agree upon. A list of all statement excluded is provided in the supplementary file (see S1 Table).

**Table 2:** Items essential for defining recovery.

Item	Round Included	Percentage Agreement
Being able to take part in life at your own pace and on your own terms rather than trying to live by standards set by society	1	93.88%
Not being dominated by thoughts of ending your life	1	90.82%
Accepting yourself for who you are, including any imperfections and giving yourself permission to be good enough	1	86.74%
Learning how to live well even with ongoing thoughts about ending your life	1	86.73%
Feeling happiness again	1	83.17%
Re-engaging with life and the social world	1	82.14%
Finding meaning or a purpose in your life; a reason to get up for each day	1	81.63%
Understanding that whilst the thoughts never completely go away, even on better days, they do become quieter again/less intense	2	91.75%
Accepting the bad things about life and the world and choosing to continue	2	89.69%
Being open to new possibilities (e.g., friends, job)	2	88.66%
Living a life guided by your values rather than your problems	2	86.60%
Recovery is a life long journey with ups and downs and twists and turns	2	85.57%
Recovery is about having the confidence to know what is right for you	2	84.54%
Not being afraid of failure or allowing fear to stop you	2	83.51%
Feeling hope, joy, and love whilst also knowing that you might consider ending your life again	2	82.47%

**Table 3:** Factors that facilitate recovery.

Item	Round Included	Percentage Agreement
Being treated by others in a kind, compassionate, empathic and non-judgmental way	1	94.90%
Developing skills to cope with difficult emotions so you don’t become overwhelmed by them	1	93.88%
Knowing when you need help and how to get it	1	93.37%
Living in a safe, stable and supportive environment	1	92.85%
Having trusting relationships with people (e.g., professionals)	1	92.35%
Having access to effective and timely support and/or treatment	1	91.83%
Developing empathy, compassion and kindness for yourself	1	91.32%
Feeling listened to and understood by others	1	90.82%
Feeling safe to talk about thoughts of ending your life	1	90.30%
Feeling understood by professionals rather than being pigeonholed as an ‘at-risk individual’	1	89.80%
Allowing yourself to celebrate the little victories, not just the big ones	1	89.79%
Being supported to develop your ability to cope with crisis	1	89.29%
(Re)building, relationships and support networks	1	89.28%
Being able to face challenges and make changes in your life	1	89.28%
Having supportive relationship with people who are there for you during the worst moments	1	89.28%
Taking small steps, having pockets of recovery	1	89.28%
Developing coping strategies that make you better at controlling your thoughts of ending your life	1	88.78%
Working towards achievable and personally meaningful goals	1	88.27%
Engaging in interests and hobbies	1	88.26%
Feeling able to be honest with others about where you are in your recovery journey	1	87.76%
Understanding your suicidal tendencies, such as learning the kind of situations where suicidal thoughts come from	1	87.76%
Not feeling under pressure to recover by service-imposed timeframes	1	87.75%
Regaining control over thoughts and feelings rather than being controlled by them	1	87.25%
Prioritising your self-care, doing things that make you feel good	1	86.74%
Having strategies for coping with life stress, for example problem-solving skills	1	86.74%
Learning to accept difficult emotions, viewing them as a part of everyday life	1	86.73%
Learning to manage important but problematic relationships (e.g., by putting boundaries in place)	1	86.23%
Not having to worry about money	1	86.22%
Expecting setbacks and picking yourself back up and carrying on	1	86.22%
Discovering the will to live	1	85.20%
Reconnecting with the self, which can involve accepting difficult life experiences	1	84.69%
Receiving psychological support, such as therapy	1	84.18%
Sorting out personal situations that are the cause of your thoughts of wanting to end your life	1	83.67%
Being part of the community and having meaningful connections with others (e.g., friends) as defined by you	1	83.67%
Feeling that others accept you as you are (your true self)	1	82.14%
Focusing on what you can control and letting go of those things you cannot	1	81.13%
Learning to recognise when and how stressors are impacting on your mental health	2	97.94%
Being able to recognise the signs that indicate that you need to take action to keep yourself safe	2	96.91%
Being part of a society that is accepting of diversity	2	96.60%
Being able to access support before you reach crisis point or make an attempt to end your life	2	95.88%
Knowing that if you experience a relapse, it does not negate all the effort and work put in to date	2	94.85%
Recognising that a sense of accomplishment does not have to come from employment; there are other places in life to find it (e.g., in daily activities).	2	93.81%
Realising that if things flare up again you can go back to get help, you are not a failure	2	93.81%
Having access to affordable psychological therapy	2	93.81%
Not comparing your progress to others	2	93.81%
Managing your expectations during tough times; there will be times where you stand still, this is ok	2	93.81%
Easier access to professional support (e.g., psychological therapy)	2	92.78%
Being offered long-term support by services and your personal network rather than just being supported in times of crisis	2	92.78%
Living in comfortable and stable housing	2	92.78%
Being patient with yourself: developing skills for managing mental health difficulties can take time	2	92.78%
Giving your body and mind time to rest after a crisis or an attempt	2	92.78%
Being supported to address difficult life experiences (abuse, bigotry, poverty, a lack of human connection)	2	92.78%
Allowing yourself to take the time needed to take a step back and make sense of your experiences	2	91.75%
Finding reasons to live for yourself, not for other people	2	91.75%
Having good sleep	2	91.75%
Being provided with support that is personalised	2	91.75%
Accepting that life can be challenging but that there are also good times	2	90.72%
Having professionals understand the context under which suicidal tendencies can occur	2	90.72%
Accepting the bad rather than papering over it with positivity	2	88.66%
Having people who give you enough space when you need to decompress	2	88.66%
Having a choice in terms of the type of talking therapy you are offered	2	88.66%
Recognising the skills that you have	2	86.60%
Working in collaboration with professionals providing you support	2	86.60%
Developing your own identity, not overshadowed by important people in your life	2	85.57%
Being given time to develop relationships with professionals supporting you	2	85.57%
Learning to distract from stressors, for example, by reading or listening to music	2	84.54%
Being able to access treatment on your own terms	2	84.54%
Continuing to practice skills developed in therapy	2	84.54%
Having a good diet	2	84.54%
Seeking out enjoyment	2	84.54%
Developing a safety plan with a supportive, non-judgmental other in case you find yourself in crisis or wanting to make another attempt	2	84.54%
Learning to communicate your emotions to others	2	84.54%
Developing an identity outside that of an attempt survivor	2	83.51%
Recognising the strength you have shown in all you have survived and managed	2	83.51%
Having supportive employers	2	83.51%
Learning to view thoughts about ending your life as short lived, like other thoughts and feelings, they won’t last forever	2	82.47%
Accepting the uncertainty of life and the future	2	82.47%
Making time for self-reflection to reconnect with and understand yourself and where you might be at	2	82.47%
Being supported by a professional (e.g., nurse, psychologist)	2	81.44%
Receiving support that takes into account your cultural and/or ethnic beliefs and/or your LGBTQ+ identity and/or disability rights (one or all of these may apply to you)	2	81.44%
Knowing that you do not have to deserve, or have a purpose, to be worthy of being alive	2	80.41%
Having hope for the future- imagining a better, brighter future for yourself	2	80.41%
Knowing the role that your life history plays in shaping your current problems, including you wanting to end your life	2	80.41%
Being supported to recognise your prospects and future opportunities	2	80.41%
Having more crisis services (beyond A&E and helplines)	2	80.41%
Learning to face difficult things rather than avoiding them	2	80.41%
Focusing on mastering daily activities (e.g., making your bed each morning) and gradually building on this	2	80.41%

**Table 4:** Factors that hinder recovery.

Item	Round Included	Percentage Agreement
When the only source of support involves being handed a list of numbers to contact in a crisis	2	84.54%
Not having your concerns taken seriously by professionals	2	83.51%
Not being able to take the time you need to recovery because of a lack of financial security	2	82.47%
When you are made to feel that you are the problem for having thoughts about suicide	2	82.47%
People becoming upset or angry with you when you talk about your suicidal thoughts	2	82.47%
Being informed that you do not meet criteria for support and then being “fobbed off”	2	82.47%
Having A&E professionals make inappropriate comments	2	80.41%
Being offered only a small number of therapy sessions	2	80.41%

A total of 15 items were identified as being essential or important in defining recovery from suicidal thoughts and behaviour, with each reaching agreement across rounds of 80% or more (Table 2). When defining recovery, being able to take part in life at your own pace and on your own terms rather than trying to live by standards set by society was viewed as important or essential by 93.88% of the panel members. On the whole, many of the items rated as essential or important in recovery appeared to reflect factors that are within the control of the individual and align with clinical (e.g. Not being dominated by thoughts of ending your life; 90.82%) as well as user-based definitions of recovery (e.g. Re-engaging with life and the social world; 82.14%).

A total of 87 statements were rated as essential or important in defining factors that facilitate recovery from suicidal thoughts and behaviours (Table 3). Being treated by others in a kind, compassionate, empathic and non-judgmental way was identified as the most essential/important factor that facilitates recovery, reaching as consensus agreement of 94.90%. When looking at the top 10 most important factors in facilitating recovery, it is evident that individual, network, service, professionals and wider systemic and societal factors are important.

When exploring factors that hinder the process of recovery (Table 4), many appear to be within the context of an individual’s experience of accessing healthcare services. For example, being supported through having a list of crisis services provided to you was viewed as essential/important in hindering recovery (84.54%).

In reviewing the list of statements across the three categories some key themes emerged, a summary of these is provide in Table 5. The most frequently occurring theme related to ‘individual’ factors. This theme included items around developing specific skills that may require support from a professional as well as items around practical things an individual can do to support their own recovery. The ‘services’ theme captured items around the need for better access to support and psychological therapy. It seemed important to participants’ recovery that support is provided continuously, rather than just at times of crisis. A number of service-related factors were also identified as important barriers to recovery; for example, being provided only with a list of numbers to contact during crisis. The ‘professionals’ theme captured items around how professionals ‘can be with’ the individual that facilitate recovery; for example, working collaboratively and taking time to understand the individual’s story. An individual’s ‘social networks’ appeared important when thinking about recovery and as such it formed one of the key themes. Again, this theme reflected the importance of interactions with others. For those with lived experience of suicidal thoughts and behaviours being seen, understood as well as having people who make it possible for them to talk about their experiences were rated as important. The final theme is ‘systemic and societal’ factors, which covered factors important in supporting recovery that centred around an individual’s living environment and the acceptance of suicidal experiences within society.

**Table 5:** Summary of themes.

Individual Factors	Service Factors	Professional Support Factors	Social Network Factors	Systemic/Societal Factors
Being able to take part in life at your own pace and on your own terms rather than trying to live by standards set by society	Having access to effective and timely support and/or treatment	Working in collaboration with professionals providing you support	Being part of the community and having meaningful connections with others (e.g., friends) as defined by you	Being part of a society that is accepting of diversity
Developing skills to cope with difficult emotions so you don’t become overwhelmed by them	Being able to access support before you reached crisis point or make an attempt to end your life	Having professionals understand the context under which suicidal tendencies can occur	Feeling listened to and understood by others	Not having to worry about money
Knowing when you need help and how to get it	Having more crisis services (beyond A&E and helplines)	Feeling understood by professionals rather than being pigeonholed as an at-risk individual	Being treated by others in a kind, compassionate, empathic and non-judgmental way	Living in a safe, stable and supportive environment

## Discussion

The current study used the Delphi expert consensus method to understand recovery from suicidal thoughts and behaviours. It is the first study which has sought to understand how recovery from suicidal thoughts and behaviours is conceptualised by those with this lived experience. Although the existing literature demonstrates that recovery is an idiosyncratic experience, the findings show that there are factors which are common across individuals. Across the two rounds, a level of consensus was reached with regards to how to define recovery, what factors support recovery and the factors that hinder this process. The findings are a significant addition to the existing literature, both in terms of providing a contribution to the growing literature that seeks to draw on the lived experience of individuals who have difficulties with suicidal thoughts and behaviours and in providing pragmatic factors to consider when developing services, interventions and prevention strategies.

The highest level of consensus regarding definitions of recovery was reached for “being able to take part in life at your own pace and on your own terms rather than trying to live by standards set by society”. This speaks to the importance of the individual being able to define their own recovery. It is about the individual defining their recovery progress and making their own choice in terms of what a meaningful life looks like to them. Indeed, one of the factors identified as important in facilitating recovery was ‘not feeling under pressure to recover by service-imposed timeframes’. Service constraints resulting from limited funding can leave individuals feeling pressured to recover and without a sense of control, further perpetuating their difficulties. There is a risk that failure to meet service-imposed recovery timeframes could leave individuals feeling defeated and burdensome, both of which are identified as risk factors for suicide [60, 61]. This indicates the importance of promoting choice, autonomy, empowerment and joint decision making when supporting individuals so that they can live a life of their own choosing.

The current study demonstrates the complexity in seeking to define recovery. Participants reached high levels of agreement for both ‘not being dominated by thoughts of ending your life’ and ‘learning how to live well even with ongoing thoughts about ending your life’. This is consistent with the dimensional recovery model proposed by Whitley and Drake [9], which bridges the gap between the competing perspectives of clinical and non-clinical definitions of recovery. Indeed, many of the factors identified here as important in defining recovery could be encompassed within the five dimensions (clinical, existential, physical, functional and social) of recovery identified by the model. Furthermore, there is overlap between the themes developed within the current study and existing models of recovery. For example, the themes of service and systemic and societal factors could be said to be similar to the external conditions (i.e., A positive culture of healing and human rights) of the Jacobson and Greenley model [8]. However, the formation of themes within the current study is unique to suicidal thoughts and behaviour and seeks to reflect the role that we all (professionals, services, significant others and wider society) can play in supporting recovery. The current findings and existing models of recovery highlight the importance of understanding how recovery is defined by the individual and then seeking to tailor support in line with this. Adopting a dimensional model of recovery may therefore be appropriate for services supporting individuals experiencing suicidal thoughts and behaviours. This approach is particularly important as recovery isn’t defined only as the absence of suicidal thoughts or behaviours.

The largest number of items were endorsed in relation to factors that help people to recovery from suicidal thoughts and behaviours. ‘Being treated by others in a kind, compassionate, empathic and non-judgmental way’ was viewed as the most essential factor in supporting individuals to recover. This points to the importance of the relational nature of suicide prevention that has been discussed within the literature [26, 27] and to the need for compassionate and empathic care [62]. It may also be reflective of the evidence which consistently demonstrates that suicide and suicide related behaviours are stigmatised within the general public [63, 64] and professional groups [65], and the need for a human response that is grounded in greater understanding.

The role of services, professionals, as well as an individual’s social network(s) and systemic factors in facilitating recovery were deemed important. This shows that there are many different ways of engaging with and supporting individuals with lived experience of suicidal thoughts and behaviours through their recovery journey. Therefore, demonstrating that each of us can play an important role and that every contact, be that with family, friends or members of the community matters. Many of the items, however, related to how the individual can facilitate their own recovery. This is consistent with what we might expect based on service user definitions of recovery which highlight the importance of the individual taking ownership of their recovery [8]. It is worth noting, however, that many of the factors (e.g., developing problem solving skills, emotional regulation) identified as important in supporting recovery, may require contact with professional staff and could be developed through engagement with psychological interventions [28].

Whilst only a small number of items were viewed as essential in acting as barriers to recovery, it is important to note that all items relating to barriers were identified by participants during round one of the research study. This demonstrated the importance of barriers in the panel members’ conceptualisation of recovery. The barriers identified related mainly to service and professional factors. Specifically, stigmatising views from staff, having needs dismissed and being provided with minimal support were all viewed as hindering recovery. These factors are worth noting, as evidence demonstrates that, for individuals presenting at accident and emergency for self-harm, negative interactions with staff and services during assessment can lead to further thoughts and acts of self-harm and discourage further help seeking and engagement [65].

### Strengths and limitations

This is the first study that has sought to develop a consensus of recovery from suicidal thoughts and behaviours. Furthermore, the use of the online survey design allowed the research team to recruit individuals across the country. Unlike previous research that has explored recovery, the Delphi method allowed us to canvas the views of a large number of individuals with lived experience of suicidal thoughts and behaviours.

A number of methodological limitations are also worth noting. Firstly, it may be that a four-month timeframe for round one was too long and might have contributed to the attrition rates we observed between rounds. Furthermore, while we tried to reach different voices through advertising the study via different platforms, we were limited in the panel members we were able to recruit. Specifically, our sample is made up of predominantly White British individuals, as such the findings are limited in terms of their generalisability to other cultures. This is of significant importance because evidence suggests that there are aspects of recovery which are unique to those belonging to Black and Minority Ethnic communities [7]. This, along with our choice of convenience sampling can limit the generalisability of the present findings. Developing an understanding of recovery with a greater focus on minoritised communities is important for future research as current models of recovery fail to make reference to conceptualisations of recovery which have emerged from non-western cultures [66]. Although, in the UK, 75.1% of those who die by suicide are men [67], the majority of our sample were women. This is consistent with excess rate of suicide attempts in women compared to men [68]. However, the bias in the sample may mean that factors which are important in supporting men to recover have not been included in the final list. Given the gender difference observed in response to suicide prevention programmes [69], future research may benefit from exploring conceptualisations separately for men and women, and then seeking to identify similarities and differences.

More recent theories of suicide [60, 70] have sought to differentiate between suicidal thoughts and behaviour and, indeed, evidence suggests that many people think about suicide without making an attempt. It may be that there are distinctions in the way that recovery is conceptualised between those who experience suicidal thoughts alone and those who go on to engage in suicidal behaviours. We were unable to draw these differences out in the current study; however, future research may wish to explore this further. Furthermore, it could be argued that the current results are biased towards those ‘early’ in their recovery and so replicating this Delphi study with those individuals with a history of suicide thoughts/behaviour but no recent (e.g., past 5 years) experiences of either may help to strengthen the generalisability of these findings. Given the large number of additional statements that were generated following round one, the study could have benefited from an additional round to further refine the final list of statements. Completing two iterations of the questionnaire is consistent with previous Delphi research [42, 71, 72], however, a future study could refine the list further by conducting a third round which drawn upon a panel that involves both lived experience individuals and professionals (e.g., researchers and clinicians). This is important as at present the clinical utility of the final list is limited, due in part to the large number of items retained across rounds of the questionnaire. Further refinement is now needed to develop a shortlist of key items that contribute most to operationalising recovery from suicidal thoughts and behaviours. Finally, it is important to acknowledge that suicidal thoughts and behaviours can occur in the context of mental health difficulties and it may be that when responding to the questionnaire these aspects of the panel members experience may have influenced or informed their responses beyond the experience of suicidal thoughts and behaviours. However, participants were given clear instructions that they should draw on their specific experiences of suicidal thoughts and behaviour when responding to the statements.

### Clinical and policy implications

There are a number of clinical implications resulting from this study. Firstly, the findings can be used to develop audit tools for evaluating how effective services are at providing recovery orientated services that are aligned with service user definitions of recovery. This is particularly important as currently services are much more focused on clinical definitions of recovery. In the UK, NHS mental health services often support individuals on a one-to-one basis. However, the current study highlights that the system (e.g., friends and family) around an individual, and the system’s response to an individual’s suicidal thoughts and behaviour, can be important facilitators and hinderances of recovery. Such information can be used by services and individual clinicians to help guide practice around the involvement of key individuals in a service user’s life and provide them with the necessary tools and knowledge to support their loved one. To date, a number of studies have demonstrated the importance of involving supportive others in all stages of an individual’s recovery journey [23, 27].

Individuals with lived experience of suicidal thoughts and behaviours agreed that being treated in a compassionate way was one of the most important facilitators of recovery, with compassion towards the self also being viewed as highly important. What appears to be expressed across many of the factors important in supporting recovery is the notion of social connection, of being seen and heard and treated with compassion and kindness during interaction with others, factors which have been observed as protective of suicidal thoughts and behaviours [73]. This demonstrates the important of ‘being with’ the individual [74]. However, when supporting individuals with suicidal thoughts and behaviours the focus is often on assessing and managing risk, which can leave individuals feeling ‘done to’, powerless and disconnected [75]. Whilst risk assessments are an important part of clinical practice and should be carried out, the current findings also demonstrate the importance of being with the individual in a compassionate and empathic way and allowing them to tell their story of how they have arrived at feeling suicidal. In practice, therefore, we might benefit from placing more of a focus than currently exists on being with the individual and understanding their story and the strengths that can emerge from this rather than immediately seeking solutions to the current crisis.

Systemic issues which cannot be directly tackled by services, such as being part of a society that is accepting, living in stable and safe housing and not having to worry about money were rated as important for recovery. Government policy that is focused on reducing stigma within the general public [64, 76] and in supporting individuals who may be financially vulnerable may therefore be needed. At a local level, healthcare services (e.g., mental health and A&E) may benefit from providing training around supporting individuals experiencing suicidal thoughts or behaviours which focuses on connection and compassion. Such training should be developed and delivered jointly with individuals with lived experience and can draw on the current study and existing literature around recovery. This ensures that factors important to those with lived experience are considered.

The existing literature on recovery from suicidal thoughts and behaviours has demonstrated the idiosyncratic nature of recovery across a number of settings and cultures. The current study allowed us to further develop this understanding by demonstrating that, whilst recovery is idiosyncratic in nature, there are common factors shared across individuals experiencing suicidal thoughts and behaviours. Furthermore, by seeking to understand the perspective of those with this lived experience, the present research was able to highlight the importance of understanding factors that hinder recovery, a factor that has seen little attention to date within the literature. The findings of the current research are an important step to better understanding recovery from suicidal thoughts and behaviour and it is hoped that future research can draw from the results presented here to further refine our conceptualisation of recovery from suicidal thoughts and behaviour. Ultimately it is hoped that this research will contribute to the development of a new ‘recovery-informed’ theory of suicide, with associated measures of recovery, that can better explain how individuals themselves, and also healthcare services, are enabling or hindering recovery. However, we recognise that there is a challenge in developing such a line of research, particularly in light of a service user movement that argues against the usefulness of the concept of recovery and highlights that it can be used to withdraw support from individuals. Furthermore, in order for such a theory to be of value, it must incorporate literature that has sought to explore recovery for individuals from the Global South. It is therefore important that future research seeks to ensure the involvement of individuals with lived experience in developing this literature further. A focus on recovery can help us better understand the risk factors for suicide by helping to identify those most at risk (i.e., many risk factors and few protective factors) and can support the development of services and interventions that are recovery focused.

### Conclusion

Despite the idiosyncratic nature of recovery, the present study demonstrated that there are factors which are shared amongst individuals when conceptualising recovery from suicidal thoughts and behaviours. Consistent with existing literature on recovery, this study demonstrates that a definition of recovery must consider both clinical and user-based definitions. Currently, a theory of recovery from suicidal thoughts and behaviours does not exist. However, our findings provide an important first step by bringing together the existing literature and showing that a theory of recovery that places lived experience at its heart must consider both factors that facilitate and hinder recovery. In considering the applications of the findings of this study, we argue that recovery from suicidal thoughts and behaviour is everyone’s responsibility. The present findings highlight the important skills that those experiencing suicidal thoughts and behaviours can be supported to develop through engagement in psychological therapies, such as CBT and third wave therapies and in their contact with professionals. At present, much of the research is focused on the role of CBT, however here we show that other approaches may be worth exploring. Furthermore, they demonstrate the important role that supportive networks play and highlight that healthcare services may benefit from working more collaboratively with significant others when providing support to individuals who are suicidal. Finally, consistent with existing literature that argues for the role of societal factors (money issues, housing) in increasing the risk of suicide, the current study demonstrates the important role that addressing these issues can play in facilitating recovery. At a fundamental level, these findings provide a helpful prompt for services looking to develop support groups for those experiencing suicidal thoughts and behaviours. In order to further strengthen the findings of the current study, we recommend that the list of statements developed here are further refined by drawing on other panels, including those comprising clinical and research professionals.

## Supporting information

S1 File A list of websites. (DOCX)

S1 Table Items that there excluded in round one and round two of the study. (DOCX)
