# Supplementary material for: Developing a consensus of recovery from suicidal ideations and behaviours: A Delphi study with experts by experience
Source: PLoS One. 2023 Sep 20;18(9):e0291377. doi: 10.1371/journal.pone.0291377 (PMC10511083; doi:10.1371/journal.pone.0291377)
Supplement: S1 File — (DOCX) [file pone.0291377.s001.docx]

**S1 File. A list of websites**

https://www.nspa.org.uk

https://www.suicideinfo.ca

https://theactionalliance.org

https://livethroughthis.org

https://www.gov.uk

https://www.mentalhealth.org.uk

https://lifelineforattemptsurvivors.org

https://talkingaboutsuicide.com

http://www.thereasons.ca/about.php

https://www.mind.org.uk

https://save.org

https://www.texasbar.com

https://www.beyondblue.org.au
