# Supplementary material for: Developing a consensus of recovery from suicidal ideations and behaviours: A Delphi study with experts by experience
Source: PLoS One. 2023 Sep 20;18(9):e0291377. doi: 10.1371/journal.pone.0291377 (PMC10511083; doi:10.1371/journal.pone.0291377)
Supplement: S1 Table — (DOCX) [file pone.0291377.s002.docx]

**S1 Table. Items that were excluded in round one and round two of the study**

| Item | Stage excluded | Percentage agreement |
| --- | --- | --- |
| Receiving practical support (e.g., with daily living tasks) from others | 1 | 65.82% |
| Being able to give back to others, such as volunteering or acting as a peer mentor | 1 | 40.81% |
| Hearing others story of recovery, hope and overcoming | 1 | 44.39% |
| Focusing on something you feel grateful for each day | 1 | 53.06% |
| Recognising that others (e.g., family) would be better off with you in their lives | 1 | 60.71% |
| Recovery can be thought of as overcoming and survival | 1 | 47.96% |
| Working together with people who promote choice and self- empowerment | 1 | 57.15% |
| Having control over your appointments with professionals rather than being expected to attended weekly appointments | 1 | 47.45% |
| Keeping thoughts about ending your life away by keeping busy with day to day activities | 1 | 42.35% |
| Finding the funny side to life to help yourself feel in control when it feels like nothing else is working | 1 | 57.14% |
| Wanting to be responsible for the people that depend on you (e.g., taking care of children) | 1 | 39.29% |
| Feeling that others need you | 1 | 48.98% |
| Disconnecting with healthcare services when you want to | 1 | 35.20% |
| (Re)connecting with religion and/or spiritual practice | 1 | 11.73% |
| Reframing your difficulties, focusing on the positives rather than emphasising the negatives | 1 | 63.27% |
| Doing meditation, mindfulness or yoga | 1 | 27.55% |
| Knowing that there are people who don’t want you to end your life | 1 | 56.12% |
| Being part of a peer support group, where people share knowledge and practical advice | 1 | 35.20% |
| Reconnecting with family and friends | 1 | 69.90% |
| Fearing the aftermath of an attempt that doesn’t end your life | 1 | 35.21% |
| Becoming more aware of religious, cultural and family beliefs towards suicide | 1 | 6.12% |
| Allowing yourself to think of the future as only a short period of time (e.g., thinking one week ahead only) | 1 | 52.55% |
| Taking medication | 1 | 46.94% |
| Having dreams that are important to you that you are yet to fulfil | 1 | 62.25% |
| Becoming aware of the likely impact that ending your life might have on others (e.g., family, friends) | 1 | 50% |
| When things are difficult being able to hold on to the belief that you have a purpose | 1 | 65.31% |
| Gaining more responsibility at work or in your private life | 1 | 27.55% |
| Recognising that others in your life would miss you if your life ended | 1 | 47.45% |
| Focusing on the here and now and not thinking about the rest of the day | 1 | 57.66% |
| Developing positive thinking- such as engaging in positive self-talk | 1 | 64.80% |
| Recognising and being supported to see what you offer to society | 1 | 58.16% |
| Knowing that others feel or have felt the same as you | 1 | 61.23% |
| Expressing emotions through hobbies (e.g., art) or writing (e.g., keeping a journal) | 1 | 62.25% |
| Making a promise to yourself or to others that you will not harm yourself | 1 | 20.40% |
| Connecting with nature | 1 | 58.67% |
| Leaving a turbulent/problematic relationship or family environment | 1 | 69.90% |
| Engaging in physical activity | 1 | 65.82% |
| Feeling that you are valued by others, such as being told how important you are to other people | 2 | 72.16% |
| Having opportunities for employment and education | 2 | 78.35% |
| Developing a personal identity separate from the difficulties that the causing you to want to end your life | 2 | 78.35% |
| Being independent and taking care of yourself | 2 | 78.35% |
| Finding positive self-regard; such as knowing that you are a good, worthwhile and effective person, with a role to play in the world | 2 | 79.38% |
| Knowing where to go when your relationship with your keyworker has broken down | 2 | 74.23% |
| Recognising that you can affect your own life situation, recovery is within your control | 2 | 79.38% |
| Having structure to your day | 2 | 78.35% |
| Continuing to experience thoughts about suicide, but no longer being afraid of them | 2 | 79.38% |
| Receiving support from A&E without fear of being sectioned | 2 | 63.92% |
| Recognising the role and impact of socio-economic or socio-political context in shaping your difficulties and your recovery | 2 | 63.92% |
| Being supported by people (personal and professional) who recognise the role and impact of socio-economic or socio-political context in shaping your difficulties and your recovery | 2 | 79.38% |
| Reducing social media time | 2 | 38.14% |
| Recovery is about getting back to how you used to be, to the person you feel you were | 2 | 21.65% |
| Learning to live in the moment | 2 | 72.16% |
| Developing your motivation | 2 | 77.32% |
| Sitting with and tolerating intense feelings rather than hiding from them using distraction | 2 | 69.07% |
| Continuing to have suicidal thoughts but not engaging in suicidal behaviour | 2 | 75.26% |
| Having a dedicated team within A&E who you could talk to about your suicidal thoughts | 2 | 53.61% |
| Learning not to fear pain, suffering or problems | 2 | 65.98% |
| Having pets | 2 | 37.11% |
| Services providing support for friends and family to help them understand the impact of mental health on all involved. | 2 | 77.32% |
| Having a dedicated team within A&E where you can receive support with your physical wounds | 2 | 58.76% |
| Recovery is about accepting that “life can suck” and that you may never find your ‘purpose’ or full happiness | 2 | 61.86% |
| Having your own designated support worker rather than being supported by whoever is available that day | 2 | 75.26% |
| Having the opportunity to develop employability skills as part of your mental health support | 2 | 53.61% |
| Recovery is about finding the minimum change necessary for you to lead a happy life | 2 | 37.11% |
| Being happier more than you are sad | 2 | 46.39% |
| Making an active choice to get up and push through each day | 2 | 71.13% |
| Offloading work | 2 | 52.58% |
| Recovery is about acceptance of suicidal thoughts rather than seeking to control them | 2 | 65.98% |
| Recognising that a lack of progress in therapy can be a reflection on the professional rather than on yourself | 2 | 57.73% |
| Recognising that you are not unfixable, it’s the treatments provided that are not meeting your needs | 2 | 70.10% |
| Having quick access to medication when you recognise that you are approaching a difficult time with your mental health | 2 | 69.07% |
| When the worst parts of your experience become a little bit better | 2 | 70.10% |
| Imagining things could be worse or have been worse instead of hoping that they get better, to feel positive about a situation | 2 | 37.11% |
| Receiving positive regard from others, not being viewed as “other” | 2 | 77.32% |
| Attending group therapy | 2 | 16.49% |
| Recognising that just coping is enough | 2 | 76.29% |
| Being told to think positively about yourself or your life when you’ve experienced so much hardship | 2 | 72.16% |
| Being supported by professionals who don’t understand your experiences | 2 | 71.13% |
| Being told by professionals that you are a failure because you view your suicidal thoughts as a coping mechanism that will be with you for your while life. | 2 | 79.38% |
| Having to spend hours in A&E in order to receive support | 2 | 71.13% |
| Being taken to hospital by police officers | 2 | 63.92% |
| When you are supported by inexperienced staff | 2 | 70.10% |
| Being pushed to engage in meditation when you have thousands of things running through your mind | 2 | 63.92% |
| Being isolated in a room (in A&E) on your own during times of crisis | 2 | 58.76% |
| Having to stay in a difficult job because it is your only source of financial security | 2 | 79.38% |
| People asking you to make a promise that you will not hurt yourself | 2 | 73.20% |
| Being told to think about the impact your decision to end your life could have on significant people in your life | 2 | 73.20% |
| When you are encouraged to revisit past trauma | 2 | 32.99% |
| Having fixed expectations of what recovery tasks should look like for you day to day | 2 | 70.10% |
| Services threatening to remove support for missing appointments | 2 | 72.16% |
| Hearing others story of recovery when you are fairly early on in your journey of recovery | 2 | 26.80% |
| Being labelled by health professionals and placed into a category | 2 | 48.45% |
| When you are pressured, by others, to engage in activities that are viewed as being important in supporting your recovery | 2 | 58.76% |
| The pressures placed on us by a capitalist society | 2 | 59.79% |
| Feeling hopelessness around climate change | 2 | 37.11% |
| Cuts made to social care | 2 | 74.23% |
| When you chose to live for another individual or a relationship | 2 | 55.67% |
| Being forced onto medication by professionals | 2 | 47.42% |
| Being “kicked out” of therapy before your (psychological) wounds have healed. | 2 | 72.16% |
| Being supported by professionals who are more concerned about the impact of your suicide on others than the pressures you are managing | 2 | 79.38% |
| Societal stigma around ‘invisible’ mental health conditions | 2 | 78.35% |
| Not being provided an appointment when in crisis | 2 | 78.35% |
| Being told to engage in psychological therapy when you don’t feel this will benefit you | 2 | 48.45% |
